# Estrogen predicts multimodal emotion recognition accuracy across the menstrual cycle

**DOI:** 10.1371/journal.pone.0312404

**Published:** 2024-10-22

**Authors:** Daisung Jang, Max Lybeck, Diana Sanchez Cortes, Hillary Anger Elfenbein, Petri Laukka

**Affiliations:** 1 Melbourne Business School, University of Melbourne, Carlton, Victoria, Australia; 2 Department of Psychology, Stockholm University, Stockholm, Sweden; 3 Olin Business School, Washington University in St. Louis, St. Louis, Missouri, United States of America; 4 Department of Psychology, Uppsala University, Uppsala, Sweden; King Saud University / Zagazig University, SAUDI ARABIA

## Abstract

Researchers have proposed that variation in sex hormones across the menstrual cycle modulate the ability to recognize emotions in others. Existing research suggests that accuracy is higher during the follicular phase and ovulation compared to the luteal phase, but findings are inconsistent. Using a repeated measures design with a sample of healthy naturally cycling women (N = 63), we investigated whether emotion recognition accuracy varied between the follicular and luteal phases, and whether accuracy related to levels of estrogen (estradiol) and progesterone. Two tasks assessed recognition of a range of positive and negative emotions via brief video recordings presented in visual, auditory, and multimodal blocks, and non-linguistic vocalizations (e.g., laughter, sobs, and sighs). Multilevel models did not show differences in emotion recognition between cycle phases. However, coefficients for estrogen were significant for both emotion recognition tasks. Higher within-person levels of estrogen predicted lower accuracy, whereas higher between-person estrogen levels predicted greater accuracy. This suggests that in general having higher estrogen levels increases accuracy, but that higher-than-usual estrogen at a given time decreases it. Within-person estrogen further interacted with cycle phase for both tasks and showed a quadratic relationship with accuracy for the multimodal task. In particular, women with higher levels of estrogen were more accurate in the follicular phase and middle of the menstrual cycle. We propose that the differing role of within- and between-person hormone levels could explain some of the inconsistency in previous findings.

## Introduction

The ability to recognize others’ emotions benefits many aspects of personal and social functioning [1]. Women generally perform better than men on emotion recognition tests, although effect sizes are usually small [2, 3]. Biologically, men and women have different levels of sex hormones (i.e., estrogen, progesterone, and testosterone [4], which likely influence development of some behavioral sex differences [5, 6]. Estrogen and progesterone fluctuate during the phases of the menstrual cycle in women, which may influence perception and behavior [7, 8] as well as mental health outcomes [9]. During the follicular phase (from the onset of menstruation to ovulation) both estrogen and progesterone levels are initially low. Estrogen markedly increases in the late follicular phase and peaks during ovulation, while progesterone remains low. During the luteal phase (following ovulation until the next menstruation) both estrogen and progesterone are generally high (e.g., [10]). These hormonal changes could influence emotion recognition. Notably, neuroimaging studies suggest that female sex hormones modulate activity in the amygdala and prefrontal cortex, which are regions important for the processing of emotions [11, 12]. Thus, researchers have proposed that sex hormone fluctuation during the menstrual cycle could influence emotion recognition accuracy (e.g., [13]). In the current study, we therefore investigated the effects of sex hormones and menstrual cycle phase on emotion recognition rates in a sample of healthy naturally cycling women.

Previous studies suggest that females may be slightly better at recognizing facial expressions during the follicular/ovulatory phase than during the luteal phase (for reviews, see [14, 15]), although these studies find mixed results. Some studies found higher overall recognition accuracy in the follicular vs. luteal phase [16–19]. In line with this group difference, Derntl et al. [16, 18] reported negative correlations between overall accuracy and progesterone levels. These effects may also vary by stimulus characteristics, such as type of emotion. Pearson and Lewis [13] reported that accuracy for fearful faces was highest during the late follicular phase compared to other cycle phases. Guapo et al. [20] reported that accuracy for angry and sad faces was higher during the follicular vs. luteal period. Some studies have also reported positive correlations between recognition accuracy and levels of estrogen, albeit with some qualifications. In Hamstra et al. [21] this effect applied only to happy faces and participants who carried a certain mineralocorticoid haplotype. In Gamsakhurdashvili et al. [22] this effect only appeared for expression stimuli from male actors.

From an evolutionary perspective, enhanced emotion recognition in the follicular phase could reflect the importance of social interaction during the most fertile period to increase mating chances [15]. Alternatively, decreased accuracy in the luteal phase could reflect heightened sensitivity to emotional cues [23] and bias toward negative emotions [24]. This may result in overperceiving potentially threatening stimuli which has a protective function for the fetus during pregnancy [16].

By contrast, other studies have reported no effects on emotion recognition of cycle phase [25–27], progesterone levels, or estrogen levels (e.g., [19, 27]). Results from some studies even go in the opposite direction as those reviewed above, with negative correlations between estrogen and recognition accuracy [20, 25]. Shirazi et al. [28] also reported no evidence that estrogen or progesterone levels predicted results on the Reading-the-Mind-in-the-Eyes test [29]. However, this test does not measure emotion recognition per se, but instead assesses the related ability of ‘theory of mind’ which focuses on reasoning about the mental states of others.

Most past research investigated the recognition of still images of facial expressions (but see [25, 27]). In real social situations, however, emotions are expressed by a combination of dynamic facial, vocal and bodily expressions [30]. Previous studies have also focused on a limited number of emotion categories, including only a single positive emotion (i.e., happiness). Such a design contrasts with the potential to communicate a wide range of both positive and negative emotions [31].

The current study introduces more ecologically valid measures of emotion recognition into the study of sex hormones and cycle phase. We used two emotion recognition tasks, both of which included dynamic expressions. Both tasks also included a wider range of emotions than in past work, including several positive emotions other than happiness. In the first task, emotions were expressed through facial expressions and speech prosody, and stimuli contained both unimodal (video only, audio only) and multimodal (audio-visual) items. The second task contained non-linguistic vocalizations—i.e., non-speech human sounds such as grunts, screams, and laughter—which are especially suited for emotional expression [32]. These two tasks were used to investigate in detail whether emotion recognition accuracy varies across three factors, namely the follicular vs. luteal phases, high vs. low estrogen, and high vs. low progesterone. We hypothesized that emotion recognition will be more accurate in the follicular vs. luteal phase. On an exploratory basis without prediction based on previous research, we did not have grounds for specific hypotheses about associations between accuracy and hormone levels [15].

## Method

### Participants and procedure

The sample consisted of 67 female individuals of reproductive age ranging from 18 to 30 years (M = 22.98, SD = 3.61) and in self-reported good health (M = 5.90, SD = 1.01, on a scale from 1 = very bad to 7 = excellent). None of the participants reported using hormonal contraceptives, being pregnant, or any history of substance abuse or mental disorders. Written informed consent was obtained prior to participation. The study was approved by the Stockholm area Regional Ethical Review Board (decision no. 2014/375-31). Participants received three movie vouchers as compensation for their voluntary participation.

A repeated measures design was used where each individual came to the lab (at Stockholm University, Sweden) on two occasions, with the aim that the interval between visits would be approximately two to three weeks. During each test occasion, the participants reported the number of days since onset of their last menses (which was used to categorize menstrual cycle phase), provided saliva samples for hormonal analyses (described below), and took part in two emotion recognition tasks (also described below). Participants also took part in other tests not reported in this paper referring to social perception of faces and economic decision making. Recruitment was conducted between August 2014 and January 2015.

In a standardized 28-day menstrual cycle, the follicular phase lasts from onset of menstruation on day 1 to ovulation on day 14, whereas the luteal phase lasts from ovulation to the onset of the next menstruation on (days 15–28) (e.g., [33]). However, individual differences in cycle length, and especially the length of the follicular phase, are common (e.g., [34, 35]). We were not able to standardize our cycle phase data onto a standard 28-day cycle, because we did not have data on participants’ cycle length (only the onset of their last menses). In our study, we thus defined the follicular phase as 1–18 days, and the luteal phase as 19 or more days, after the onset of a participant’s latest menstruation. This was based on a study of more than 600,000 real-world menstrual cycles [34], which reported that the average length of the follicular phase among 18-30-year-old women from Sweden, UK, and USA was around 18 days.

The average number of days since last menses was 8.74 (SD = 5.33, range = 1–18) in the follicular condition and 24.50 (SD = 4.39. range = 19–35) in the luteal condition. Thirty-five participants were in the follicular phase and 25 in the luteal phase during the first test occasion, with similar proportions during the second test occasion (follicular, N = 38; luteal, N = 21). During one of their test sessions, 7 participants did not report the number of days since last menses so it was not possible to categorize their responses into phases for that session. If cycle phase information was missing for a particular testing session, the data were excluded from analysis because there was no way to incorporate such data in computing phase differences. We found that 21 participants were in the same cycle phase during both test occasions. To maximize power, we utilized all data that were possible to use in the analyses, and only dropped observations with missing data for 4 participants where neither estrogen or progesterone could be determined for both test sessions. These criteria resulted in N = 63 for the mixed model analyses (also described below).

### Measures

#### Hormonal measures

We measured sex hormone levels via saliva samples from each participant at both test occasions. The samples were sent to a professional reference laboratory (Dresden LabService GmbH) for analysis of levels of estrogen (estradiol) and progesterone. Saliva samples were collected by passive drool using commercially available collection devices (SaliCaps, IBL International, Hamburg, Germany) and were frozen immediately after collection and stored at -20°C. Following all data collection, samples were shipped overnight on dry ice to the lab for single determination of estradiol and progesterone levels. After thawing, the saliva samples were centrifuged at 3,000 rpm for 5 min, which resulted in a clear supernatant of low viscosity. Salivary concentrations of estradiol and progesterone were measured using commercially available chemiluminescence immunoassays with high sensitivity (IBL International, Hamburg, Germany). The laboratory that performed the analyses reported that the intra- and inter-assay coefficients of variation were below 11%.

As expected (e.g., [10]), progesterone levels were significantly lower in the follicular phase (M = 156.29 pg/ml, SD = 132.56) than in the luteal phase (M = 309.17 pg/ml, SD = 248.74); independent samples *t*-test with separate variance estimates, *t*(61.32) = 3.84, *p* < .001, *d* = 0.77. Estradiol levels were also lower in the follicular (M = 6.72 pg/ml, SD = 3.88) vs. the luteal (M = 7.73 pg/ml, SD = 5.70) phase, but this difference did not reach statistical significance, *t*(71.40*)* = 1.06, *p* = .29, *d* = .21. We also checked the normality of the estrogen and progesterone distributions using the Shapiro-Wilks test. The test suggested both estrogen (W = 0.74, *p* < .001) and progesterone (W = 0.77, *p* < .001) had non-normal distribution. The non-significant difference in estradiol levels across phases could be attributable to the non-normal distributions. Thus, we conducted bootstrapped *t*-tests with 2,000 replications, as it is a method to account for the non-normal distribution [36]. These tests showed the same pattern as the non-bootstrapped tests, i.e., a significant difference for progesterone (95% CI [82.50, 235.11], *p* < .001) and non-significant difference for estrogen (95% CI [-0.64, 3.21], *p* = .25).

#### The multimodal emotion recognition test (ERAM)

The Emotion Recognition in Multiple Modalities (ERAM) test [37] measured the ability to recognize emotions accurately from dynamic facial, vocal, and multimodal expressions. The test consists of stimuli from the Geneva Multimodal Emotion Portrayal corpus (GEMEP) [30]. This database consists of video clips of emotion expressions which are portrayed by 10 professional actors in interaction with a professional theatre director. Each of the ERAM videos shows close-up views of the actor’s face and upper torso while he or she is speaking pseudo-linguistic sentences (e.g., “nekal ibam soud molen!”). Thus, these videos include facial, vocal and also some bodily cues to the expressed emotion. Pseudo-linguistic sentences were used in order to avoid semantic content to confound results. ERAM includes 72 unique items which convey 12 emotions with both positive and negative valence (happiness, interest, pleasure, pride, relief, hot anger, anxiety, despair, disgust, panic fear, irritation and sadness). Items were presented in fixed order in 3 blocks: 24 items with video only, 24 items with audio only, and 24 items with multimodal (both audio and video) stimuli. Each emotional state appeared twice in each block. This method allowed for separate assessment of emotion recognition from visual, auditory and multimodal stimuli. The duration of the video clips varied between 1-5s, and sound level was normalized separately for each actor. After each item, participants viewed a list of the 12 intended emotion expressions and selected the alternative they thought best captured the emotion conveyed by the item. The ERAM test took a total of approximately 15–20 minutes to complete and was always presented as the first task during each testing occasion.

#### The non-linguistic vocalization test (VENEC)

The ability to accurately recognize non-linguistic vocalizations (affect bursts) was assessed using stimuli from the Vocal Expressions of Nineteen Emotions across Cultures (VENEC) corpus. VENEC is a database consisting of vocal emotion expressions which are depicted by 100 actors from 5 different English-speaking nations [38]. The task included 18 different emotions: 9 positive emotions (affection, amusement, happiness, interest, sexual lust, pride, positive surprise, relief, and serenity) and 9 negative emotions (anger, contempt, disgust, distress, fear, guilt, negative surprise, sadness, and shame). The vocalizations consisted of human non-speech sounds such as sighs, breathing sounds, crying, hums, grunts, laughter, and shrieks. Each emotion was represented by 12 vocal stimuli, leading to a total number of 108 items. Participants judged positive and negative emotions in two separate tasks, and the order of the tasks (and items within the tasks) was randomized for each participant. After presentation of an item, participants selected the emotion that they thought best captured the emotion conveyed by the vocalization (the alternatives they could choose among were the intended 9 positive or 9 negative emotions). In order to reduce the discrepancy between sounds that would have been too loud (e.g., screams) or too quiet (e.g., whispers), the sound levels of the vocal stimuli were normalized. The VENEC test of vocalizations took approximately 30 minutes to complete, and it was always presented as the second task during each testing occasion.

### Data processing and analysis

Data were analyzed using the *R* statistical programming language (version 4.2.0) and multilevel modeling was conducted using the *lme4* package (version 1.1–29) [39]. These analyses predicted emotion recognition accuracy based on the following variables: menstrual cycle phase (follicular, luteal), modality (visual, auditory, multimodal, only applicable for the ERAM test), individual emotions (12 emotions for the ERAM, and 9 negative and 9 positive emotions for the VENEC), test occasion (occasion 1, occasion 2), and levels of estrogen and progesterone.

Analyzing longitudinal data allows disambiguating two types of effects, one that occurs within a person, and another that occurs between people [40]. For example, we reviewed evidence that suggested greater emotion recognition accuracy during the follicular/ovulatory phase compared to the luteal phase, which implies a within-person phenomenon. However, cross-sectional data cannot provide evidence for such patterns. There may be two people, one who has a high level of recognition at the follicular/ovulatory phase and another who has a low level of recognition at the luteal phase. While this implies differences across phases (i.e., a between-person difference), it does not imply that the first person will have low levels at the luteal phase and the second will have a high level of recognition at the follicular/ovulatory phase. Using a multilevel approach with longitudinal data allowed decomposition of within- vs. between-person effects [41]. This is because longitudinal data contains information about both within- and between-person effects. Formally, within-person effects refer to individual variation in hormones and is estimated with a variable that subtracts a person’s mean level of hormone from their level of hormone at any given phase (i.e., person-centered). Between-person effects refer to person-level differences in hormones and is estimated with a variable that subtracts a person’s mean level of hormone from the grand mean of hormone level (i.e., person-mean-centered). Conceptually, within-person effects examine how a higher or lower level of hormone than could be expected for an individual at a particular moment is associated with accuracy, whereas between-person effects examine if having high or low levels of hormones is associated with accuracy. In practice, both between- and within-person effects are likely in hormone data. Women vary considerably in their levels of estrogen and progesterone [42], with one estimate suggesting that 46% of variability in progesterone is attributable to within-person sources of variation and 54% of variability is attributable to between-person sources of variation [43].

All models included the intercept and person-centered levels of estrogen and progesterone as random effects; thus, all models were built on the assumption that each subject has a different intercept and that their response to estrogen and progesterone could vary randomly. We also chose multilevel analyses because they can handle the non-independence of variables in present study (e.g., the dependency between cycle phase and test occasion), and allow one to model this dependence between variables in the data (see e.g., [44]). In additional analyses, we also tested for a quadratic (i.e., U-shaped) relationship between number of days since last menstruation and emotion recognition accuracy. This was achieved by subtracting the average days since last menstruation from the reported day and squaring that difference score. That score was then used as a predictor of recognition accuracy.

Emotion recognition accuracy measures were calculated using Wagner’s [45] *unbiased hit rate* (Hu). Hu is an accuracy measure that takes into consideration the joint probability of both the simple hit rate and the differential accuracy by multiplying these two probabilities with each other. The simple hit rate is calculated by dividing the number of times a certain emotion is correctly selected with the total frequency of that emotion. This is then multiplied with the differential accuracy, which results from dividing the number of times a certain emotion is correctly selected with the total frequency that the participant has responded with that same emotion (see [45] for more details). Hu is an unbiased measure of recognition accuracy because it merges the proportions of both response frequency and stimulus frequency. Values for Hu range from 0 to 1, and a score of 1 indicates both that all stimuli of an emotion were correctly classified, and the respective emotion was never misclassified as another emotion.

It is not possible to calculate Hu values for an individual participant’s judgments of an individual stimulus. We calculated Hu values for individual emotions for both the ERAM and VENEC tests. However, for the ERAM test, we did not calculate Hu values for individual emotions separately for each presentation modality, because the test only includes two items per emotion per modality. Following Cortes et al. [46], for the ERAM test, we instead calculated Hu values for each participant and a) each presentation modality (across all emotions) and b) each emotion (across all presentation modalities).

Upon examining the data, we observed outliers in the level of hormones. For outliers that were more than 3SD from the mean, we replaced them with a value that equaled 3SD from the mean. The pattern of results conducted with variables with and without such treatment of outliers did not substantively affect the pattern of results. Our threshold for significance was *p* < .05.

## Results

### Cycle phase differences and hormones for the ERAM test

Table 1 shows the results from four linear mixed effects models using emotion recognition accuracy (Hu) on the ERAM test as the outcome measure. Presentation modality (Models 1 and 2) and specific emotions (Models 3 and 4) appear in separate models, as detailed below.

**Table 1:** Mixed effect models of emotion recognition accuracy for dynamic multimodal expressions (ERAM).

	Accuracy (Hu)			
	Model 1	Model 2	Model 3	Model 4
Testing occasion	.19***	.20***	.12***	.12***
Cycle phase (1 = follicular, 0 = luteal)	-.01		-.02	
Day of cycle		.07		.05
Days squared		-.02		-.01
Estrogen, within-person	-.44***	.13	-.28***	.08†
Estrogen, between-person	.56***	.50***	.53***	.47***
Progesterone, within-person	.18	-.14	.11	-.08†
Progesterone, between-person	.04	.14	.03	.13
Audio modality	-.21***	-.21***		
Multimodal modality	.36***	.36***		
Anxiety			-.50***	-.50***
Despair			-.45***	-.45***
Disgust			-.24***	-.24***
Fear			-.40***	-.40***
Happiness			-.29***	-.29***
Interest			-.31***	-.31***
Irritation			-.34***	-.34***
Pleasure			-.14***	-.14***
Pride			-.30***	-.30***
Relief			-.18***	-.18***
Sadness			-.48***	-.48***
Estrogen, within-person * Cycle phase	.45***		.29***	
Progesterone, within-person * Cycle phase	-.20		-.14†	
Estrogen, within-person * Days squared		-.33**		-.21***
Progesterone, within-person * Days squared		.14		.08
Constant	.00	.00	.00	.00
Observations	357	357	1,428	1,428
Log Likelihood	210.11	186.19	328.48	304.67
Akaike’s Information Criterion	-384.22	-334.38	-602.96	-553.33
Bayesian Information Criterion	-314.42	-260.70	-460.83	-405.94

Note. † *p* < .10

* *p* < .05

** *p* < .01

*** *p* < .001, standardized coefficients reported. The reference category for the emotions was anger. *N* = 63.

#### Presentation modality

**Fig 1:**
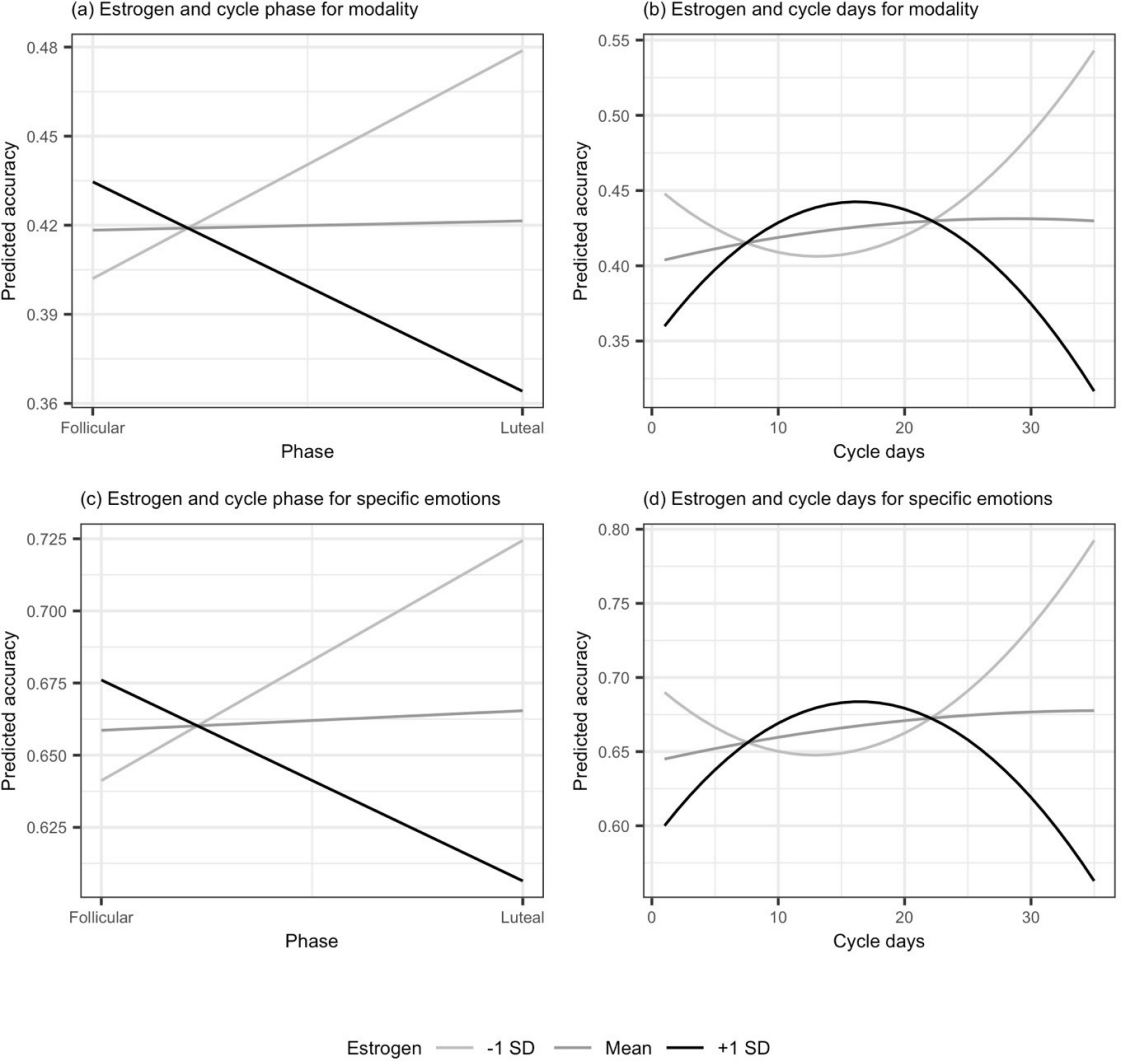
Interactions between estrogen (within-person), cycle phase, and cycle days in predicting emotion recognition accuracy (ERAM task). (A) Interaction between estrogen levels and cycle phase shown when accuracy is distinguished via presentation modality. In the follicular phase, the +1 SD line shows that accuracy is higher for women who have higher levels of estrogen and the -1 SD line shows accuracy is lower for women who have lower levels of estrogen. (B) Illustration of the interaction between estrogen levels and the days squared variable when accuracy is distinguished via presentation modality. Accuracy is highest in the middle of cycle for women who have higher levels of estrogen (+1 SD line). (C) Interaction between estrogen levels and cycle phase shown when accuracy is distinguished via specific emotion. In the follicular phase, the +1 SD line shows that accuracy is higher for women who have higher levels of estrogen and the -1 SD line shows accuracy is lower for women who have lower levels of estrogen. (D) Illustration of the interaction between estrogen levels and the days squared variable when accuracy is distinguished via specific emotion. Accuracy is highest in the middle of cycle for women who have higher levels of estrogen (+1 SD line). Accuracy levels are generally higher when distinguished by specific emotion vs. presentation modality because accuracy for the reference category (anger) is higher than for all other emotions.

Model 1 of Table 1 shows that, by contrast with the hypothesis, the coefficient for cycle phase was not significant and there was no difference in recognition accuracy between the follicular (*M* = .432, 95% CI [.408, .457]) and luteal phases (*M* = .435, 95% CI [.404, .467], *d* = .030). Note that all reported mean values in the results section represent the estimated marginal means. However, indicators for the auditory and multimodal modalities were significant compared to the baseline visual modality. Post hoc multiple comparisons (*t*-tests with Bonferroni corrections) showed that accuracy with multimodal expressions (*M* = .520, 95% CI [.487, .554]) was significantly higher than with visual expressions (*M* = .420, 95% CI [.387, .454], *d* = .950), which in turn was higher than with auditory expressions (*M* = .361, 95% CI [.328, .395], *d* = .562), with all of these comparisons significant. There was a significant main effect of test occasion that reflected a training effect between the first test occasion (*M* = .409, 95% CI [.381, .436]) and the second test occasion (*M* = .459, 95% CI [.433, .484], *d* = .473). We observed a significant negative coefficient for within-person levels of estrogen but a positive between-person effect—that is, generally high levels of estrogen increase accuracy, but higher-than-average levels of estrogen at any given time decrease accuracy. There was a moderating effect whereby within-person estrogen interacted with the follicular phase indicator, such that women with higher levels of estrogen were more accurate when tested in the follicular phase (see Fig 1A). A similar interaction between progesterone and the follicular phase indicator did not reach significance.

As secondary analyses, we tested for a potential quadratic relationship between the days of the cycle and accuracy. In this Model 2, Table 1, neither day of cycle nor the quadratic term were significant. The indicator variables that represented the auditory and multimodal modalities were significant compared to the baseline visual modality, which is similar to the prior set of analyses. The effect of testing occasion was also significant, with greater accuracy in the second occasion than the first. This again represents a training effect for participants engaging in the same task on two occasions. The estimated marginal means for the secondary analyses are reported in S1 Table. Between-person estrogen predicted greater accuracy, which indicates that generally having higher levels of estrogen predicts greater accuracy. We observed a significant interaction between within-person levels of estrogen and the days-squared variable (Fig 1B), which suggests that accuracy is highest in the middle of the cycle for women with higher levels of estrogen. The interaction between progesterone and the days-squared variable was not significant.

#### Specific emotions

In addition to the effects of modality, we examined the influence of specific emotions. In Model 3, Table 1, the coefficient for cycle phase was not significant, indicating no difference in recognition accuracy between the follicular (*M* = .430, 95% CI [.407, .454]) and luteal (*M* = .437, 95% CI [.407, .467], *d* = .039) phases. Accuracy varied across the various emotional categories. Table 2 shows a matrix of comparisons between emotions, with Bonferroni adjusted *p*-values. Anger (which was also the reference category in the analyses) was the most recognized emotion and anxiety the least recognized. There was a significant effect of testing occasion, again representing a training effect, with lower accuracy in the first (*M* = .409, 95% CI [.383, .436]) vs. the second occasions (M = .458, 95% CI [.433, .483], *d* = .276). Both within-person and between-person levels of estrogen were significant predictors of accuracy. In this case, within-person levels of estrogen negatively predicted accuracy and between-person levels of estrogen positively predicted accuracy, as in Model 1, Table 1. Also similar to the preceding analyses, within-person estrogen interacted with the follicular phase indicator, such that women with higher levels of estrogen at the follicular phase were more accurate (see Fig 1C). A similar interaction between progesterone and follicular phase indicator was not significant.

**Table 2:** Accuracy on the ERAM task.

	Anger	Irritation	Disgust	Despair	Pride	Anxiety	Interest	Happiness	Fear	Pleasure	Relief	Sadness
Anger	[.662]	< .0001	< .0001	< .0001	< .0001	< .0001	< .0001	< .0001	< .0001	0.000	< .0001	< .0001
Irritation	0.255	[.408]	0.101	0.019	1.000	< .0001	1.000	1.000	1.000	< .0001	< .0001	0.000
Disgust	0.182	-0.072	[.480]	< .0001	1.000	< .0001	1.000	1.000	< .0001	0.044	1.000	< .0001
Despair	0.338	0.083	0.156	[.324]	0.000	1.000	0.001	< .0001	1.000	< .0001	< .0001	1.000
Pride	0.228	-0.027	0.046	-0.110	[.434]	< .0001	1.000	1.000	0.054	< .0001	0.005	< .0001
Anxiety	0.378	0.123	0.196	0.040	0.150	[.284]	< .0001	< .0001	0.092	< .0001	< .0001	1.000
Interest	0.237	-0.017	0.055	-0.101	0.009	-0.141	[.425]	1.000	0.212	< .0001	0.001	< .0001
Happiness	0.219	-0.035	0.037	-0.119	-0.009	-0.159	-0.018	[.443]	0.013	< .0001	0.022	< .0001
Fear	0.305	0.050	0.122	-0.033	0.077	-0.073	0.067	0.085	[.358]	< .0001	< .0001	1.000
Pleasure	0.104	-0.150	-0.078	-0.234	-0.124	-0.273	-0.133	-0.115	-0.200	[.558]	1.000	< .0001
Relief	0.137	-0.118	-0.045	-0.201	-0.091	-0.241	-0.100	-0.082	-0.168	0.033	[.525]	< .0001
Sadness	0.359	0.104	0.177	0.021	0.131	-0.019	0.122	0.140	0.054	0.255	0.222	[.303]

Note. The upper triangle represents Bonferroni adjusted *p*-values of pairwise comparisons. The diagonal represents mean accuracy (unbiased hit rate). The lower triangle represents difference score of comparisons.

We repeated the analyses using the quadratic term in place of the follicular phase indicator. In Model 4, Table 1, neither day of cycle nor days squared were significant. A significant coefficient of test occasion was observed, reflecting a training effect between the first and second occasion, as in the prior analyses (see S1 Table for estimated marginal means). Within-person level of estrogen marginally predicted accuracy and between-person estrogen significantly predicted accuracy. We again observed a significant interaction between estrogen and the quadratic term, as shown in Fig 1D. An interaction between progesterone and the quadratic term was not significant.

### Cycle phase differences and hormones for the VENEC test

Table 3 shows the results from four linear mixed effects models using emotion recognition accuracy (Hu) from non-linguistic vocalizations as the outcome measure. Positive emotions (Models 1 and 2) and negative emotions (Models 3 and 4) were included in separate models, as detailed below. Selected estimated marginal means from the models reported below are available in S1 Table.

**Table 3:** Mixed effect models of emotion recognition accuracy for non-linguistic vocalizations (VENEC).

	Accuracy (Hu)			
	Model 1	Model 2	Model 3	Model 4
Testing occasion	.05*	.05†	.03	.03
Cycle phase (1 = follicular, 0 = luteal)	.01		.02	
Day of cycle		.02		-.02
Days squared		-.05		-.04
Estrogen, within-person	-.07	.06	-.08*	.03
Estrogen, between-person	.25	.24	.59**	.56**
Progesterone, within-person	.05	-.10*	.07	-.03
Progesterone, between-person	-.08	-.02	.02	.09
Amusement	.14***	.14***		
Happiness	.20***	.20***		
Interest	.39***	.39***		
Lust	.57***	.57***		
Pride	.17***	.17***		
Positive surprise	.35***	.35***		
Relief	.74***	.74***		
Serenity	.41***	.41***		
Contempt			-.24***	-.24***
Disgust			-.09***	-.09***
Distress			-.51***	-.51***
Fear			-.32***	-.32***
Guilt			-.71***	-.71***
Negative surprise			-.40***	-.40***
Sadness			-.13***	-.13***
Shame			-.78***	-.78***
Estrogen, within-person * Cycle phase	.10		.10*	
Progesterone, within-person * Cycle phase	-.09		-.06	
Estrogen, within-person * Days squared		-.06		-.05
Progesterone, within-person * Days squared		.07		.07†
Constant	.00	.00	.00	.00
Observations	1,035	1,035	1,035	1,035
Log Likelihood	468.05	447.12	455.07	432.56
Akaike’s Information Criterion	-888.11	-844.24	-862.15	-815.12
Bayesian Information Criterion	-769.50	-720.69	-743.54	-691.56

Note. † *p* < .10

* *p* < .05

** *p* < .01

*** *p* < .0001, standardized coefficients reported. The reference category was serenity and anger for positive and negative emotions, respectively. N = 63.

#### Positive emotions

Starting with positive emotions, Model 1, Table 3, showed a non-significant coefficient for cycle phase, with no difference between recognition accuracy in the follicular (*M* = .322, 95% CI [.298, .346]) vs. luteal (M = .317, 95% CI [.288, .345], *d* = .041) phase. However, there was a significant effect of testing occasion, with lower accuracy in the first (*M* = .309, 95% CI [.283, .334]) vs. second (*M* = .330, 95% CI [.306, .353], *d* = .153) occasion. Neither estrogen nor progesterone predicted accuracy. There was considerable variability in the accuracy of individual emotions, with Bonferroni corrected pairwise comparisons shown in Table 4.

**Table 4:** Accuracy on the positive emotions on the VENEC task.

	Affection	Amusement	Happiness	Interest	Lust	Pride	Positive surprise	Relief	Serenity
Affection	[.115]	< .0001	< .0001	< .0001	< .0001	< .0001	< .0001	< .0001	< .0001
Amusement	-0.086	[.201]	1.000	< .0001	< .0001	1.000	< .0001	< .0001	< .0001
Happiness	-0.125	-0.039	[.240]	< .0001	< .0001	1.000	< .0001	< .0001	< .0001
Interest	-0.239	-0.153	-0.114	[.354]	< .0001	< .0001	1.000	< .0001	1.000
Lust	-0.350	-0.264	-0.225	-0.111	[.465]	< .0001	< .0001	< .0001	< .0001
Pride	-0.105	-0.019	0.020	0.134	0.245	[.220]	< .0001	< .0001	< .0001
Positive surprise	-0.219	-0.133	-0.094	0.020	0.131	-0.114	[.334]	< .0001	1.000
Relief	-0.460	-0.374	-0.335	-0.221	-0.110	-0.355	-0.241	[.575]	< .0001
Serenity	-0.254	-0.168	-0.129	-0.015	0.096	-0.149	-0.035	0.206	[.369]

Note. The upper triangle represents Bonferroni adjusted *p*-values of pairwise comparisons. The diagonal represents mean accuracy (unbiased hit rate). The lower triangle represents difference score of comparisons.

As with the ERAM analyses, we examined if there was a quadratic relationship between cycle phase days and accuracy. In Model 2, Table 3, the day of cycle, days squared, and estrogen did not predict accuracy. However, within-person levels of progesterone negatively predicted accuracy. There was also a marginally significant coefficient for testing occasion, with lower accuracy in the first occasion compared to the second.

#### Negative emotions

**Fig 2:**
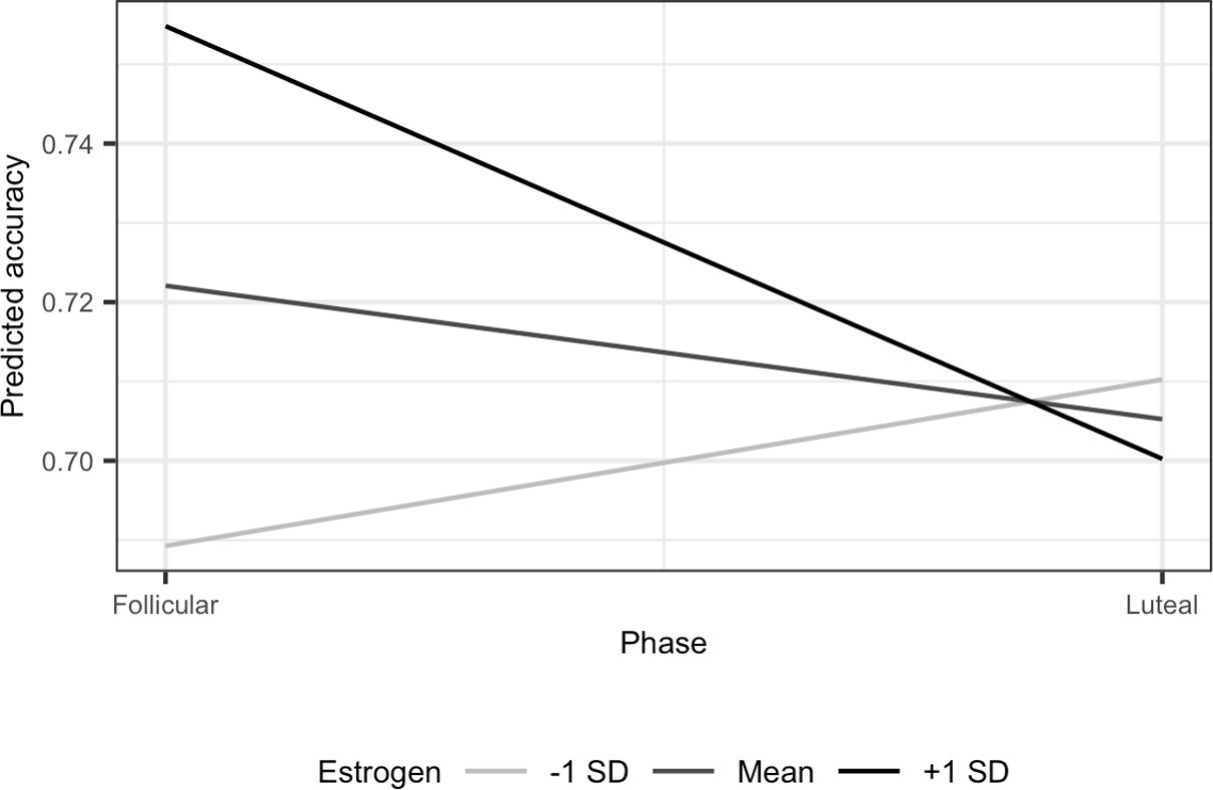
Interaction between estrogen and cycle phase in predicting emotion recognition accuracy for negative emotions (VENEC task). Interaction between estrogen levels and cycle phase shown when accuracy is distinguished via type of negative emotions. In the follicular phase, the +1 SD line shows that accuracy is higher for women who have higher levels of estrogen and the -1 SD line shows accuracy is lower for women who have lower levels of estrogen. As in Fig 1, accuracy levels are high when distinguished by specific emotion because accuracy for the reference category (anger) is higher than for all other emotions.

In Model 3, Table 3, the coefficient for cycle phase was not significant, with no significant difference between the follicular (*M* = .441, 95% CI [.417, .465]) and the luteal (*M* = .430, 95% CI [.401, .459], *d* = .078) phases. There was no significant difference in accuracy between the first (*M* = .428, 95% CI [.402, .454]) vs. second (*M* = .444, 95% CI [.420, .468], *d* = .115) occasions. Within-person estrogen negatively predicted while between-person positively predicted accuracy. In addition, within-person estrogen significantly interacted with the follicular phase indicator, suggesting higher accuracy in the follicular phase for women with higher levels of estrogen (see Fig 2). Neither progesterone nor interactions between progesterone and cycle phase predicted accuracy. There was considerable variability in the accuracy of individual emotions, with Bonferroni corrected pairwise comparisons shown in Table 5.

**Table 5:** Accuracy on the negative emotions on the VENEC task.

	Anger	Contempt	Disgust	Distress	Fear	Guilt	Negative surprise	Sadness	Shame
Anger	[.723]	< .0001	0.002	< .0001	< .0001	< .0001	< .0001	< .0001	< .0001
Contempt	0.192	[.531]	< .0001	< .0001	0.004	< .0001	< .0001	0.000	< .0001
Disgust	0.075	-0.117	[.648]	< .0001	< .0001	< .0001	< .0001	1.000	< .0001
Distress	0.414	0.222	0.339	[.310]	< .0001	< .0001	< .0001	< .0001	< .0001
Fear	0.262	0.071	0.188	-0.151	[.461]	< .0001	0.015	< .0001	< .0001
Guilt	0.577	0.385	0.502	0.163	0.314	[.147]	< .0001	< .0001	0.075
Negative surprise	0.327	0.135	0.252	-0.087	0.065	-0.249	[.396]	< .0001	< .0001
Sadness	0.108	-0.084	0.033	-0.306	-0.155	-0.469	-0.220	[.616]	< .0001
Shame	0.633	0.441	0.558	0.219	0.371	0.056	0.306	0.525	[.090]

Note. The upper triangle represents Bonferroni adjusted *p*-values of pairwise comparisons. The diagonal represents mean accuracy (unbiased hit rate). The lower triangle represents difference score of comparisons.

As with the analyses above, we further examined if there was a quadratic relationship between the cycle phase days and accuracy. In Model 4, Table 3, there were no significant coefficients for the day of cycle term, days squared term, within-person estrogen, nor progesterone. However, between-person levels of estrogen predicted accuracy as in the prior analyses, and the interaction between progesterone and days squared was marginally significant.

### Model assumptions and robustness tests

To increase confidence in the pattern of data observed, we checked model assumptions across all of the analyses conducted. Examination of residuals through Q-Q plots revealed that none of the models suffered from non-normally distributed residuals. The models were not influenced by multicollinearity, with all variance inflation factors below 5. We also conducted analyses to examine if estimates we obtained could be misleading due to power issues. Thus, we examined Type S and M errors in our findings [47]. Type S errors are defined as the probability that the estimate is of the incorrect sign, and Type M errors are defined as the amount that the observed effect size exaggerates the likely true effect size. We examined Type S and M errors for all significant hormone coefficients and interaction terms that involve hormones, and observed that the highest likelihood of a Type S error was less than .0001 and that the highest estimate of Type M error did not exceed 1.5. This provides confidence in the accuracy of estimates.

We also conducted additional tests to examine the robustness of the result. One limitation of how we defined the follicular and luteal phases is that they overlap with the midcycle phase. This can make it difficult to distinguish the qualitatively different hormone profiles in the follicular and luteal phases. To observe more distinct differences between the follicular and luteal phases, we conducted analyses that should remove midcycle observations for the majority of participants by excluding days 15–22 of the cycle (see S2 and S3 Tables). The pattern of results remained largely the same. The main exceptions concerned within-person levels of estrogen. The coefficients for within-person levels of estrogen increased and became significant when predicting ERAM while controlling for a continuous measure of days of the cycle, and presentation modality (Model 2, S2 Table) and specific emotions (Model 4, S2 Table). However, when predicting negative emotions on the VENEC when controlling for cycle phase (Model 3, S3 Table), the coefficient for within-person estrogen and the coefficient for the interaction between estrogen and the follicular phase indicator changed from significant to marginally significant despite small increases in magnitude. Progesterone estimates also remained largely similar, with one exception. When predicting positive emotions on the VENEC with a continuous measure of days of the cycle (Model 2, S3 Table), within-person progesterone no longer predicted accuracy, although the coefficient increased slightly and remained marginally significant.

## Discussion

Our first main finding was the lack of significant associations between menstrual cycle phase and the ability to recognize emotions from dynamic multimodal expressions (ERAM) or non-linguistic vocalizations (VENEC). This was the case in both the main analyses and in additional robustness tests.

Our second main finding was a significant positive association between recognition accuracy and between-person estrogen level, yet a negative association between accuracy and within-person estrogen level. Taken together, this suggests that having generally higher estrogen may increase accuracy, whereas a higher-than-average level of estrogen at any given time may instead decrease it. These results were consistent in analyses involving positive and negative emotions in the ERAM test and for negative emotions for the VENEC test. In terms of robustness, the negative associations between accuracy and within-person estrogen occurred only in the models that controlled for cycle phase, and not in the models that included a continuous measure of days of the cycle, whereas the positive associations between accuracy and between-person estrogen appeared across both types of models.

Our third main finding was consistent interactions between cycle phase and within-person level of estrogen for the ERAM task, which suggests that women with higher levels of estrogen were more accurate when tested in the follicular phase.

Finally, our fourth main finding concerned a quadratic relationship between days of the menstrual cycle and emotion recognition accuracy. While we did not observe significant coefficients for the quadratic term, there were consistent significant interaction coefficients for within-person levels of estrogen across analyses for the ERAM test. This suggests an inverse-U like relationship where higher level of estrogen was associated with greater accuracy during the middle of the menstrual cycle, but with lower accuracy toward the beginning and the end of the cycle.

In addition to the main findings, our analyses detailed how emotion recognition varied as a function of task characteristics such as presentation modality and emotion. Because these findings did not relate to cycle phase or hormone levels, they are not discussed in depth, although they replicate well what has been observed in previous studies using the same emotion recognition tasks (e.g., [37, 46]). We also noted a significant negative association between within-person progesterone level and recognition accuracy in one analysis for the VENEC test. While this is consistent with some previous studies [16, 18], we hesitate to emphasize this finding due to concerns about robustness, given that results for progesterone tended to vary across analyses in contrast with consistency for analyses of estrogen.

Contrary to several previous studies [13, 16–20], but in line with others [25–28], we did not observe menstrual cycle phase differences on emotion recognition accuracy. We suggest that the mixed results regarding cycle phase differences could result, at least in part, from variability in the method used to define and measure cycle phases in different studies (see [28]). Whereas some studies (like ours) have only categorized participants into two broad categories (follicular and luteal), other studies have instead attempted more fine-grained classifications (e.g., early follicular, ovulatory, luteal; see e.g., [20, 48]). If the effects of sex hormones vary continuously across the menstrual cycle, then more detailed categorization would be preferable because it would, for example, allow one to detect effects that occur specifically during ovulation. Luteinizing hormone tests could also be used to validate cycle phases (e.g., [49, 50]). Another limitation of our study, and several others, is the use of the forward method—that is, counting days from the onset of the last menses forward to the day of testing—to categorize cycle phase. It has been suggested that this method is prone to errors, and more reliable ways of obtaining self-reports of cycle phase have been proposed [49, 51]. We suggest that future studies could combine reliable measures of cycle phase with measurement of sex hormones to achieve a deeper understanding of how emotion recognition accuracy varies as a function of cycle phases. Alternatively, studies could also dispense with the categorization of cycle phases, and instead use days of the menstrual cycle as a continuous variable, akin to what we attempted in our analyses on a possible quadratic relationship between cycle day and emotion recognition.

A relatively novel feature of our study was that we assessed associations between both within- and between-person hormone levels and emotion recognition accuracy (see also [27]). Interestingly, the coefficients pointed in different directions with positive associations for between-person levels of estrogen, but negative associations for within-person levels (see e.g., Fig 1). This pattern (higher estrogen levels on the aggregate being beneficial, but higher-than-usual estrogen being detrimental at any one time) is called Simpson’s paradox and has been observed in multiple contexts. In one classic example, Bickel and colleagues [52] discussed the paradox in terms of admission rates at a university. While it may appear on the aggregate that male applicants were admitted at a higher rate than female applicants, the pattern reversed when examining admission rates by department—women were more likely to be admitted than men at the level of the department (see [53] for a detailed discussion). In the case of sex hormones and emotion recognition, some previous studies have reported positive associations between estrogen levels and emotion recognition accuracy (e.g., [21, 22]), whereas others have reported negative associations (e.g., [20, 25]). In light of our findings, we suggest that such mixed findings could result, at least partly, from design choices and analysis methods, where between- and within-person designs could potentially lead to different conclusions.

We also observed significant interactions that suggest the associations between estrogen and recognition accuracy may vary across the phases and days of the menstrual cycle. These interactions appeared most consistently for the ERAM test, and showed that women with higher estrogen levels had higher accuracy in the follicular phase and in the middle of the menstrual cycle. Such interactions may have also potentially contributed to the mixed findings in previous studies. Considering that estrogen levels vary the most during the follicular phase, with a peak during ovulation but relatively stable values during the luteal phase (e.g., [10]), the observed interactions could reflect increased emotion recognition accuracy closer to ovulation (see [13]).

Our findings regarding estrogen should be interpreted with caveats. All findings should be regarded as tentative until replicated, and further research is needed especially to understand better how between-person and within-person estrogen levels may have different associations with emotion recognition. Our observations do not allow us to draw conclusions about causality and further research is needed to establish potential causal roles for estrogen in enhancing or decreasing emotion recognition ability. Such studies could, for example, compare groups of women that use hormonal vs. non-hormonal contraceptives at several occasions across the menstrual cycle, which would allow some experimental control of hormone levels (e.g., [28, 48]). We also note that the validity of the immunoassay method to measure salivary estrogen and progesterone, which has been used in many studies including ours, has recently been criticized [54]. Replication efforts could thus seek additional techniques for hormone measurement such as liquid chromatography-mass spectrometry. Finally, the study was not pre-registered and a pre-registered replication attempt could ensure the robustness of the findings.

Replication efforts could systematically investigate the impact of task characteristics, given that previous work often finds associations with estrogen only in some conditions but not others (e.g., only for some emotions) (e.g., [13, 20, 21]). Previous studies have usually measured emotion recognition based on a small number of emotions appearing in still pictures of facial expression. In contrast, we measured recognition of a wide range of emotions using dynamic multimodal expressions and non-linguistic vocalizations. We argue that the latter approach provides a more ecologically valid measure of emotion recognition ability, as it manifests in daily social interactions (e.g., [30]). Given the large number of emotions included in our tasks, the most reliable measures included all together rather than examining them each separately, and further investigation of possible emotion-specific effects using more ecologically valid stimuli remains an exciting topic for future research.

The small sample sizes (typically around 20–40 participants, but see [27, 28]) and cross-sectional designs employed in previous studies may have caused problems with statistical power [49, 50, 51]. In our study, by contrast, we used a different type of design in which most participants were tested twice at different phases. Although repeated measurement does increase power, it can also lead to learning effects with improved performance between the two test occasions, and these effects can make it more difficult to assess the possible associations between cycle phase and hormones. Learning effects appeared especially for the ERAM test in our study (see also [55]). We suggest that future studies could develop tasks and designs that minimize learning effects while still benefiting from the advantages of within-subjects measurement.

To conclude, the current study expanded upon previous research on sex hormones, cycle phase and emotion recognition by using dynamic multi-modal stimuli of a wide range of positive and negative emotions. The main positive finding was that level of estrogen was associated with emotion recognition accuracy, albeit not in a straight-forward way. Between-person estrogen was positively associated with accuracy, within-person estrogen was negatively associated with accuracy, and associations with estrogen further varied across the cycles and days of the menstrual cycle. Analyses did not demonstrate consistent significant cycle phase differences for emotion recognition across analyses. These findings contribute another piece in the puzzle to the growing literature on the psychological correlates of sex hormones. More research is needed to understand the possible causal mechanisms of how sex hormones may affect psychological functioning factors that include emotion recognition, and such knowledge could have important implications for well-being and social functioning.

## Supporting information

S1 Table Selected estimated marginal means from models reported in text. (PDF)

S2 Table Mixed effect models of emotion recognition accuracy for dynamic multimodal expressions (ERAM) with midcycle observations removed. (PDF)

S3 Table Mixed effect models of emotion recognition accuracy for non-linguistic vocalizations (VENEC) with midcycle observations removed. (PDF)
