# Supplementary material for: Estrogen predicts multimodal emotion recognition accuracy across the menstrual cycle
Source: PLoS One. 2024 Oct 22;19(10):e0312404. doi: 10.1371/journal.pone.0312404 (PMC11495617; doi:10.1371/journal.pone.0312404)
Supplement: S1 Table — (PDF) [file pone.0312404.s001.pdf]

S1 Table. Selected estimated marginal means from models reported in text.

Model	Variable	Estimate	95% CI LL	95% CI UL
Table 1 Model 1	Follicular	0.432	0.408	0.457
	Luteal	0.435	0.404	0.467
	Audio	0.361	0.328	0.395
	Video	0.420	0.387	0.454
	Audio + video	0.520	0.487	0.554
	Test 1	0.409	0.381	0.436
	Test 2	0.459	0.433	0.484
Table 1 Model 2	Audio	0.363	0.330	0.395
	Video	0.422	0.390	0.454
	Audio + video	0.522	0.489	0.554
	Test 1	0.409	0.384	0.434
	Test 2	0.462	0.436	0.487
Table 1 Model 3	Follicular	0.430	0.407	0.454
	Luteal	0.437	0.407	0.467
	Test 1	0.409	0.383	0.436
	Test 2	0.458	0.433	0.483
Table 1 Model 4	Test 1	0.410	0.385	0.434
	Test 2	0.461	0.436	0.485
Table 3 Model 1	Follicular	0.322	0.298	0.346
	Luteal	0.317	0.288	0.345
	Test 1	0.309	0.283	0.334
	Test 2	0.330	0.306	0.353
Table 3 Model 2	Test 1	0.313	0.291	0.336
	Test 2	0.332	0.309	0.354
Table 3 Model 3	Follicular	0.441	0.417	0.465
	Luteal	0.430	0.401	0.459
	Test 1	0.428	0.402	0.454
	Test 2	0.444	0.420	0.468
Table 3 Model 4	Test 1	0.430	0.407	0.453
	Test 2	0.447	0.424	0.470

Note. Estimated marginal means are reported. Means reported may be influenced by their involvement in interactions. 95% CI LL = 95% Confidence Interval Lower Limit. 95% CI UL = 95% Confidence Interval Upper Limit
