# Supplementary material for: Estrogen predicts multimodal emotion recognition accuracy across the menstrual cycle
Source: PLoS One. 2024 Oct 22;19(10):e0312404. doi: 10.1371/journal.pone.0312404 (PMC11495617; doi:10.1371/journal.pone.0312404)
Supplement: S2 Table — (PDF) [file pone.0312404.s002.pdf]

S2 Table. Mixed effect models of emotion recognition accuracy for dynamic multimodal expressions (ERAM) with midcycle observations removed.

	Accuracy (Hu)			
	Model 1	Model 2	Model 3	Model 4
Testing occasion	.21**	.23***	.14***	.14***
Cycle phase (1 = follicular, 0 = luteal)	.04		.01	
Day of cycle		.03		.03
Days squared		.04		.03
Estrogen, within-person	-.42**	.39***	-.29**	.25***
Estrogen, between-person	.58***	.50**	.52***	.44**
Progesterone, within-person	.26†	-.17	.15	-.09
Progesterone, between-person	.03	.19	.04	.17
Audio modality	-.20***	-.20***		
Multimodal modality	.38***	.38***		
Anxiety			-.50***	-.50***
Despair			-.44***	-.44***
Disgust			-.24***	-.24***
Fear			-.41***	-.41***
Happiness			-.28***	-.28***
Interest			-.32***	-.32***
Irritation			-.34***	-.34***
Pleasure			-.14***	-.14***
Pride			-.28***	-.28***
Relief			-.18***	-.18***
Sadness			-.47***	-.47***
Estrogen, within-person * Cycle phase	.49***		.35***	
Progesterone, within-person * Cycle phase	-.30†		-.19†	
Estrogen, within-person * Days squared		-.52***		-.34***
Progesterone, within-person * Days squared		.16		.08
Constant	.00	.00	.00	.00
Observations	285	285	1,140	1,140
Akaike Inf. Crit.	-260.8	-217.9	-450.6	-408.3
Bayesian Inf. Crit.	-195.0	-148.5	-314.5	-267.2

Note: † $p < .10$, * $p < .05$, ** $p < .01$, *** $p < .001$, standardized coefficients reported. AIC: Akaike's Information Criterion. BIC: Bayesian Information Criterion. The reference category for the emotions was anger.
