# Supplementary material for: Estrogen predicts multimodal emotion recognition accuracy across the menstrual cycle
Source: PLoS One. 2024 Oct 22;19(10):e0312404. doi: 10.1371/journal.pone.0312404 (PMC11495617; doi:10.1371/journal.pone.0312404)
Supplement: S3 Table — (PDF) [file pone.0312404.s003.pdf]

S3 Table. Mixed effect models of emotion recognition accuracy for non-linguistic vocalizations (VENEC) with midcycle observations removed.

	Accuracy (Hu)			
	Model 1	Model 2	Model 3	Model 4
Testing occasion	.07*	.06†	.00	.01
Cycle phase (1 = follicular, 0 = luteal)	.00		.01	
Day of cycle		.03		.00
Days squared		-.04		-.04
Estrogen, within-person	-.09	.06	-.10†	.06
Estrogen, between-person	.26	.25	.69**	.69***
Progesterone, within-person	.10	-.12†	.07	-.04
Progesterone, between-person	-.12	-.05	.08	.13
Amusement	.13***	.13***		
Happiness	.22***	.22***		
Interest	.37***	.37***		
Lust	.58***	.58***		
Pride	.17***	.17***		
Positive surprise	.35***	.35***		
Relief	.74***	.74***		
Serenity	.40***	.40***		
Contempt			-.25***	-.25***
Disgust			-.08**	-.08**
Distress			-.50***	-.50***
Fear			-.32***	-.32***
Guilt			-.71***	-.71***
Negative surprise			-.39***	-.39***
Sadness			-.14***	-.14***
Shame			-.77***	-.77***
Estrogen, within-person * Cycle phase	.11		.12†	
Progesterone, within-person * Cycle phase	-.15		-.05	
Estrogen, within-person * Days squared		-.08		-.09
Progesterone, within-person * Days squared		.08		.08
Constant	.00	.00	.00	.00
Observations	819	819	819	819
Akaike Inf. Crit.	-660.3	-614.7	-628.8	-583.9
Bayesian Inf. Crit.	-547.3	-497.0	-515.8	-466.2

Note. † $p < .10$, * $p < .05$, ** $p < .01$, *** $p < .001$, standardized coefficients reported. AIC: Akaike's Information Criterion. BIC: Bayesian Information Criterion. The reference category was serenity and anger for positive and negative emotions. respectively.
